# Age Differences in Becoming COVID Long-Haulers and in Post-Acute Sequelae of SARS-CoV-2

**DOI:** 10.1093/geroni/igab046.054

**Published:** 2021-12-17

**Authors:** Qiao Wu, Eileen Crimmins

**Affiliations:** University of Southern California, Los Angeles, California, United States

## Abstract

People who have had COVID-19 can suffer from the continuation of Post-Acute Sequelae of SARS-CoV-2 (PASC), also known as “long COVID”, for months after infection. Understanding PASC is important for treatment, care, and projecting future health of the population. Since older adults are at higher risk of severe illness and consequences from COVID, we hypothesize that they are more likely to become COVID long-haulers and report more symptoms at the time of diagnosis and three months after. We use a nationally representative sample of adults from the Understanding America Study COVID-19 Survey, from March to December 2020, to estimate the prevalence of long COVID and identify the most common long-term symptoms and how they vary by age. We use multilevel models to examine the determinants of symptom count and change over time. Among the 608 people with a COVID diagnosis, 83 (13.7%) aged over 65; almost half (47.9%) reported symptoms three months after diagnosis; the proportion did not differ across age groups. The most common symptoms were fatigue (25.0%), runny/stuffy nose (18.9%), body aches (16.4%), sneezing (15.1%), and headache (13.6%). These symptoms were consistent across age groups, while people aged 65 and older reported significantly less cough (χ2=3.96; P=0.05) and headache (χ2=4.24; P=0.04) compared to their younger counterparts. Neither the mean at the time of the diagnosis nor the rate of change of the symptom count varied across age groups. Our analyses suggest that age is not a significant determinant of PASC symptom count or becoming a COVID long-hauler.

